# Impact of ICD-9-CM to ICD-10-CM coding transition on trauma hospitalization trends among young adults in 12 states

**DOI:** 10.1186/s40621-021-00298-x

**Published:** 2021-01-25

**Authors:** Yuri V. Sebastião, Gregory A. Metzger, Deena J. Chisolm, Henry Xiang, Jennifer N. Cooper

**Affiliations:** 1 Center for Surgical Outcomes Research and Center for Innovation in Pediatric Practice, Abigail Wexner Research Institute at Nationwide Children’s Hospital, Columbus, OH USA; 2 Present address: Division of Global Women’s Health, School of Medicine, University of North Carolina, Chapel Hill, NC USA; 3 Department of Surgery, College of Medicine, The Ohio State University, Columbus, OH USA; 4 Center for Population Health and Equity Research and Center for Innovation in Pediatric Practice, Abigail Wexner Research Institute at Nationwide Children’s Hospital, Columbus, OH USA; 5 Department of Pediatrics, College of Medicine, The Ohio State University, Columbus, OH USA; 6 Division of Health Services Management & Policy, College of Public Health, The Ohio State University, Columbus, OH USA; 7 Center for Pediatric Trauma Research and Center for Injury Research and Policy, Abigail Wexner Research Institute at Nationwide Children’s Hospital, Columbus, OH USA; 8 Division of Epidemiology, College of Public Health, The Ohio State University, Columbus, OH USA

**Keywords:** ICD-10-CM, External cause of injury, Traumatic brain injury, Injury severity score

## Abstract

**Background:**

We aimed to estimate the impact of the International Classification of Diseases, Tenth Revision, Clinical Modification (ICD-10-CM) coding transition on traumatic injury-related hospitalization trends among young adults across a geographically and demographically diverse group of U.S. states.

**Methods:**

Interrupted time series analyses were conducted using statewide inpatient databases from 12 states and including traumatic injury-related hospitalizations in adults aged 19–44 years in 2011–2017. Segmented regression models were used to estimate the impact of the October 2015 coding transition on external cause of injury (ECOI) completeness (percentage of hospitalizations with a documented ECOI code) and on population-level rates of injury-related hospitalizations by nature, intent, mechanism, and severity of injury.

**Results:**

The transition to ICD-10-CM was associated with a drop in ECOI completion in the transition month (− 3.7%; *P* < .0001), but there was no significant change in the positive trend in ECOI completion from the pre- to post-transition periods. There were significant increases post-transition in the measured rates of hospitalization for traumatic brain injury (TBI), unintentional injury, mild injury (injury severity score (ISS) < 9), and injuries caused by drowning, firearms, machinery, other pedestrian, suffocation, and unspecified mechanism. Conversely, there were significant decreases in October 2015 in the rates of hospitalization for assault, injuries of undetermined intent, injuries of moderate severity (ISS 9–15), and injuries caused by fire/burn, other pedal cyclist, other transportation, natural/environmental, and other specified mechanism. A significant increase in the percentage of hospitalizations classified as resulting from severe injury (ISS > 15) was observed when the general equivalence mapping maximum severity method for converting ICD-10-CM codes to ICD-9-CM codes was used. State-specific results for the outcomes of ECOI completion and TBI-related hospitalization rates are provided in an online supplement.

**Conclusions:**

The U.S. transition from ICD-9-CM to ICD-10-CM coding led to a significant decrease in ECOI completion and several significant changes in measured rates of injury-related hospitalizations by injury intent, mechanism, nature, and severity. The results of this study can inform the design and analysis of future traumatic injury-related health services research studies that use both ICD-9-CM and ICD-10-CM coded data.

**Level of evidence:**

II (Interrupted Time Series)

**Supplementary Information:**

The online version contains supplementary material available at 10.1186/s40621-021-00298-x.

## Background

Starting on October 1, 2015, all U.S. hospitals and providers covered by the Health Insurance Portability and Accountability Act (HIPAA) were required to use the tenth revision of the International Classification of Diseases, Tenth Revision, Clinical Modification (ICD-10-CM) coding system for administrative and billing data (National Center for Health Statistics 2015). The introduction of the ICD-10-CM coding system was expected to cause some disruptions in the continuity of morbidity trends derived from administrative data, as was the case after the 1999 transition from ICD-9 to ICD-10 coding for mortality data (Fenton and Benigni 2014; Gibson et al. 2016). Observed trends in the coding of traumatic injury, the leading cause of death and disability among young people in the U.S., were expected to be particularly impacted by the transition in coding systems, because the number of injury diagnosis codes increased from approximately 2600 in ICD-9-CM to 43,000 in ICD-10-CM, and the number of external cause of injury (ECOI) codes, formerly referred to as E-codes in ICD-9-CM, increased from approximately 1300 to 7500 (National Center for Injury Prevention and Control 2009; Injury Surveillance Workgroup 9 2016).

To facilitate injury research and surveillance after the coding system transition, the Centers for Disease Control and Prevention (CDC) proposed new ICD-10-CM injury surveillance frameworks to replace the widely used ICD-9-CM ECOI and injury diagnosis (nature and body region of injury) matrices (Hedegaard et al. 2016; Annest et al. 2014; Barell et al. 2002). Using the 2014 proposed ECOI matrix for ICD-10-CM data, Slavova et al. reported on the impacts of the transition to ICD-10-CM on measured injury hospitalization trends in the state of Kentucky (Slavova et al. 2018). This study found that some changes could be explained by the new ICD-10-CM-specific coding guidelines. For example, the percentage of injuries coded as unintentional increased, consistent with ICD-10-CM guidelines requiring intent to be coded as accidental whenever it is unknown or unspecified. Other changes reflected structural differences between the two coding systems. For example, the rate of hospitalizations coded as being due to poisoning increased immediately after the coding transition as a result of poisoning being captured solely through injury diagnosis codes that combine information on both nature and intent of injury in ICD-10-CM (Annest et al. 2014; Slavova et al. 2018). Recently, in December 2019, the CDC published a final ECOI matrix, with modifications from the proposed matrix that were aimed at improving continuity between trends derived from ICD-10-CM and ICD-9-CM (Hedegaard et al. 2019). To date, no studies have used the CDC’s final ECOI matrix to examine the effects of the transition to ICD-10-CM on rates of hospitalizations coded as traumatic-injury related in a multi-state cohort. Importantly, the extent to which the transition has impacted apparent traumatic injury hospitalization trends by severity or nature of injury, as determined from injury diagnosis codes, has also not been described.

The objective of this study was to examine the effects of the ICD-9-CM to ICD-10-CM coding transition on apparent trends in traumatic injury-related hospitalizations among young adults in the U.S., in order to inform future studies using hospital discharge data that span the coding transition. We examined the impact of the coding transition on the completion of ECOI coding and on population-level rates of traumatic injury-related hospitalizations by coded intent, mechanism, and severity of injury. Finally, we also examined changes in population-level rates of hospitalizations coded as related to traumatic brain injury (TBI), one of the most common causes of injury-related hospitalization and morbidity in young adults.

## Methods

### Study design and data sources

We conducted an interrupted time series (ITS) analysis using discharge data on traumatic injury-related hospitalizations that occurred between January 2011 and December 2017 in 12 U.S. states. ITS is a quasi-experimental design that is useful for examining the impact of well-defined policy changes, interventions or events, on population-level outcomes (Slavova et al. 2018; Paixão et al. 2019; Penfold and Zhang 2013; Salemi et al. 2019). Data were aggregated at the month level, based on the date of discharge. Months were classified into two time periods: before (Jan 2011-Septermber 2015), and after (October 2015–December 2017) the transition to ICD-10-CM coding.

Hospital discharge data were obtained from the 2011–2017 Healthcare Cost and Utilization Project (HCUP) State Inpatient Databases (SID) of the following 12 states: Arkansas, Colorado, Georgia, Iowa, Kansas, Kentucky, Nevada, New Jersey, New Mexico, North Carolina, Oregon, and Rhode Island. The SID contain inpatient discharge data from acute care community hospitals in participating states, encompassing all patients regardless of payer (HCUP Central Distributor Availability of Databases 2020). The availability of the SIDs and the availability and quality of some of the data elements in the SIDs vary by state and year (HCUP Central Distributor Availability of Databases 2020). At the time of analysis, 2017 was the most recent year for which SID data were available for most states. The 12 included states were selected because their SIDs contained the discharge month variable throughout the entire study period and because their SIDs have high quality patient race/ethnicity data, which are required for the funded research project for which the data were purchased. This study was approved by the Nationwide Children’s Hospital Institutional Review Board with a waiver of written informed consent. The investigators also signed data use agreements with the Agency for Healthcare Research and Quality for the use of the SID. For each of the 12 included states, annual population estimates of the number of residents aged 19–44 years in each state were obtained from the U.S. Census Bureau in order to calculate population-level rates.

### Study population

To select the study population, we initially identified non-elective hospitalizations by in-state residents aged 19–44 years that listed a primary diagnosis of injury. Young adults aged 19–44 years were selected because we acquired the SID for a funded research project that focuses on trauma care and outcomes in this age group, in whom traumatic injury is the leading cause of mortality. The list of included injury diagnoses, which was based on the National Trauma Data Standard (NTDS), is provided in the Additional file, Table A1.1 (The American College of Surgeons 2020). Only hospitalizations with an included injury diagnosis listed in the primary diagnosis field were included. Compared to the CDC’s ICD-9-CM and ICD-10-CM surveillance case definitions for injury hospitalizations, the NTDS case selection criteria do not include primary diagnoses related to poisoning and toxic effects, late effects of injuries, superficial injuries and effects of foreign bodies, other and unspecified effects of external causes, injuries complicating pregnancy, childbirth and the puerperium, or prosthetic fractures around a prosthetic joint (The American College of Surgeons 2020; Hedegaard and Johnson 2019; Injury Surveillance Workgroup 2003).

### Outcome measures

The following outcome measures were examined as either monthly percentages or monthly rates per 100,000 persons: ECOI code completion, percentage of hospitalizations by injury intent and severity, rates of hospitalization by mechanism (cause) of injury, and rate of traumatic brain injury (TBI)-related hospitalization. The numerator for rate outcomes was the monthly number of hospitalizations in the study population, and the denominator was the midyear population estimate of the number of residents aged 19–44 in each state. ECOI completion was defined as the percentage of included injury hospitalizations that had a valid ECOI code. To evaluate changes in the distribution of hospitalizations by injury intent, we calculated separate measures for the percentages of traumatic injury hospitalizations that listed codes for unintentional, intentional self-harm, assault, and undetermined injury intent based on the definitions provided in the CDC’s ICD-9-CM and ICD-10-CM ECOI matrices (Hedegaard et al. 2019; Centers for Disease Control and Prevention 2018). Only the first listed ECOI code was used to define injury intent when more than one ECOI code was documented.

To evaluate changes in injury severity, we calculated the percentages of hospitalizations for mild, moderate, and severe injury based on an estimated injury severity score (ISS) of < 9, 9–15, and > 15 respectively. Injury severity scores were calculated using the ICD Program for Injury Categorization in R statistical software, version 0.1.0 (ICDPIC-R) (Clark et al. 2018). The original ICDPIC was developed for use with ICD-9-CM codes, while ICDPIC-R allows the use of both ICD-9-CM and ICD-10-CM codes, with options to calculate injury severity directly from ICD-10-CM codes (based on diagnosis-specific mortality estimates from the National Trauma Data Bank) or indirectly by first mapping ICD-10-CM codes to ICD-9-CM codes using the Centers for Medicare & Medicaid Services’ General Equivalence Mapping (GEM) tables. We used the GEM mapping method to calculate injury severity because this method is recommended for data that contains a mix of both ICD-9-CM and ICD-10-CM codes. However, because some ICD-10-CM injury diagnosis codes map to multiple ICD-9-CM codes with different injury severities, ICDPIC-R provides the option of assigning the ICD-9-CM code with either the higher (GEMmax) or lower (GEMmin) severity. We report results for injury severity outcome measures calculated using both the GEMmax method and the GEMmin method. Hospitalizations with burn injuries were excluded from the analyses of injury severity because ICDPIC does not classify burn injuries. To date, new versions of ICDPIC-R have not been made available in the public domain, and two major issues identified with version 0.1.0 of the software are that only ICD-10-CM codes containing 7 characters are recognized and only codes with a 7th character of “A” are recognized (Issues. ICDPICR 2020). In addition, version 0.1.0 uses only the 2016 GEM. Injury codes that are not recognized are assigned a severity of 0. Our team revised and updated the ICDPIC-R software code to allow recognition of codes with a 7th character of “B” or “C”, codes that contain fewer than 7 characters, and new codes that were included in the fiscal years 2017 and 2018 updates of the GEM. We report results from analyses using both the publicly available ICDPIC-R version 0.1.0 and our team’s updated version of ICDPIC-R.

Rates of hospitalizations by mechanism of injury included separate measures based on the common mechanisms recommended for reporting injury data by the CDC in their ICD-9-CM and ICD-10-CM ECOI matrices (Hedegaard et al. 2019; Centers for Disease Control and Prevention 2018). Poisoning-related hospitalizations are not part of the NTDS inclusion criteria and therefore were not included in this study. We also did not evaluate specific injury mechanism categories that were new in the ICD-10-CM ECOI matrix and not included in the ICD-9-CM ECOI matrix (e.g. motor vehicle-nontraffic).

Finally, we evaluated rates of TBI-related hospitalizations, overall and separately by subcategories. TBI-related hospitalizations were identified using the diagnosis codes available in the CDC’s ICD-9-CM Barell injury diagnosis matrix and the proposed TBI surveillance definitions from the CDC’s ICD-10-CM injury diagnosis matrix (Hedegaard et al. 2016; Barell et al. 2002). All records in the study population that contained one or more diagnosis codes in the list of TBI diagnoses (whether primary or secondary) were considered TBI-related hospitalizations. The ICD-9-CM code for head injury, unspecified (959.01) was not included in our definition of TBI. Although medical coders do sometimes use code 959.01 to code for unspecified intracranial injuries, this code is not intended to be assigned to TBI cases and is not included in the ICD-9-CM Barell matrix definition of TBI (Hedegaard et al. 2016; Barell et al. 2002). We used the proposed surveillance TBI subcategories from the CDC’s ICD-10-CM injury diagnosis matrix to define TBI subcategories for analysis, and we identified equivalent ICD-9-CM codes for these subcategories using the Barell matrix and the 2017 GEM (Hedegaard et al. 2016; Barell et al. 2002). Table A1.2 lists the diagnosis codes used to define each TBI subcategory. Of all possible types of injuries, we focused on TBI because it is commonly targeted for research and surveillance, and it is associated with substantial morbidity and mortality in the young adult population. Analyses evaluating trends in injury intent and injury mechanisms were restricted to hospitalizations with an available ECOI code.

### Statistical analysis

Segmented regression models with autoregressive errors were used to estimate changes in outcome measures attributable to the transition to ICD-10-CM coding (Penfold and Zhang 2013; The AUTOREG Procedure 2014). Data were aggregated by month for the segmented regression analyses. For each outcome measure, the segmented regression model included the following covariates: month (a continuous variable with values 1–84, which measured the monthly trend in the outcome measure up until the transition to ICD-10-CM), time period (a dichotomous indicator for the ICD-10-CM coding system, which measured the immediate level change in the outcome measure in October 2015), and month after ICD-10-CM transition (a continuous variable with values 1–27, which measured the change in the monthly trend after the coding system transition). We report model estimates for immediate level changes as well as the changes in monthly trends or the estimated monthly trends after transition. The latter were estimated by summing the model estimates for the monthly trend pre-transition and the change in monthly trend after the transition (Linden 2015). Because time series data are prone to autocorrelation and seasonality, all models were estimated accounting for autoregressive errors (Penfold and Zhang 2013). Backwards selection was used to select the optimal order of the autoregressive model, with an initial seasonal lag of 12 months for the full model and with *p* < 0.05 used as the criterion to retain autoregressive parameters. For all outcomes in the main analysis, model assumptions and fit were assessed by reviewing diagnostic plots of predicted versus observed values, residuals and standardized residuals, white noise probabilities, autocorrelation functions, and partial autocorrelation functions (The AUTOREG Procedure 2014; Chvosta and Little 2009). We conducted several sensitivity analyses to test the robustness of our findings. We repeated all segmented regression models after removing the data point for October 2015 to assess whether observed immediate impacts of the transition on outcomes remained during the second month after the transition. We also refit all segmented regression models for injury intent and injury mechanism after removing data from December 2017, as we observed a substantial drop in ECOI code completion during this final month of the study period due to a sudden drop in ECOI coding at one large trauma center. We also repeated all analyses after restricting the study population to patients from 8 states with high (> 90%) ECOI code completion throughout the entire study period (Arkansas, Colorado, Georgia, Iowa, Kansas, North Carolina, New Jersey, and Oregon). Additional analyses stratified by the presence of a state-level mandate for ECOI reporting for injury-related hospitalizations were also planned, but these analyses were not performed because we observed a poor correlation between the reported presence of a state ECOI mandate and high ECOI completion in that state (Centers for Disease Control and Prevention 2008). States with such mandates accounted for only half of the states with high ECOI completion rates throughout the study period (Centers for Disease Control and Prevention 2008). Although between-state comparisons were not the primary focus of this study, we report separate models for ECOI code completion and TBI-related hospitalization rates for each of the 12 states in the study in Additional file 2. Finally, as an ad hoc, descriptive analysis we reviewed changes in frequencies of hospitalizations by injury intent across each injury mechanism category between September and October 2015, in order to further elucidate potential reasons for our injury intent findings.

## Results

The study population included a total of 274,439 traumatic injury related hospitalizations among young adults. The distribution of hospitalizations by state is described in Table A1.3.

### External cause of injury completion

**Fig. 1:**
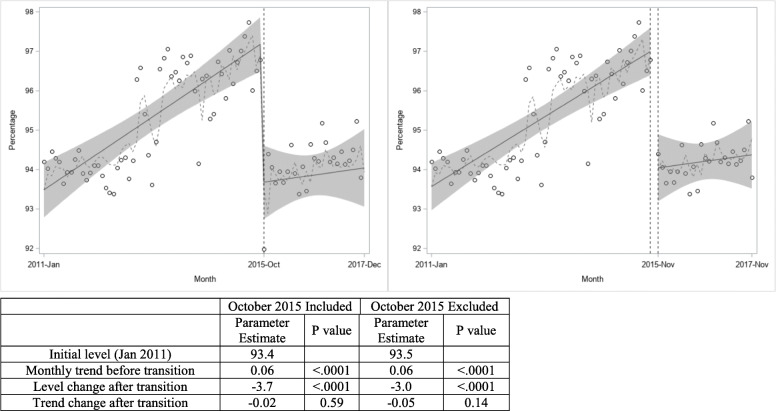
Effects of the transition to ICD-10-CM on ECOI completion for traumatic injury related hospitalizations among young adults. The immediate drop in ECOI completion following the transition to the ICD-10-CM coding guidelines in October 2015 remained beyond the first month after the transition (right plot excludes Oct. 2015 data point). There was no change in the pre- and post-transition trend

Upon initial review of trends in ECOI completion, we detected a large drop in ECOI code completion in December 2017, the last month of the study period. This drop was attributed to a lack of any ECOI codes for over 98% of hospitalizations at one large trauma center during this month. The data point for December 2017 was therefore excluded from all subsequent analyses of ECOI completion. Across all states, the percentage of hospitalizations with an ECOI code was 93.4% in January 2011, and increased significantly at a monthly rate of 0.06% in the period before ICD-10-CM. As shown in Fig. 1, in October 2015, the first month after the transition to ICD-10-CM, there was a level drop of 3.7% in ECOI completion (from 97.1% in September to 93.4% in October, 2015; *p* < 0.0001). However, the positive trend in ECOI completion after the transition was similar to that observed during the period before the transition (*p* = 0.59 for trend change). After exclusion of the October 2015 data point, there was still a level drop in ECOI completion of 3.0% (*p* < 0.0001; Fig. 1). In analyses stratified by state, the immediate drop in ECOI completion associated with the October 2015 transition was significant in 7 of the 12 states, with level drops ranging from − 2.8% in New Jersey (*p* < 0.0001) to − 22.8% in Nevada (*p* < 0.0001) (Additional file 2, Figures A2.1–2 and Table A2.1). In analyses restricted to 8 states that had consistently high (> 90%) ECOI completion throughout the entire study period, the immediate drop in ECOI completion associated with the October 2015 transition was smaller but remained significant at − 1.9% (*p* < 0.0001; Table A1.4). Model diagnostic plots showed good fit after correction for autocorrelation, with less than 5 data points having standardized residual values between 2 and 3. Findings from model fit diagnostic plots for all subsequent outcomes were similar to those seen for ECOI completion.

### Injury intent

**Fig. 2:**
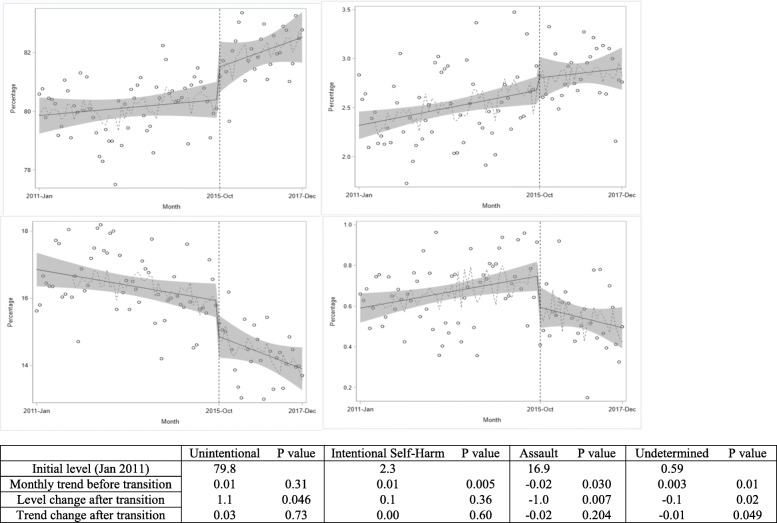
Effects of the transition to ICD-10-CM on traumatic injury related hospitalizations among young adults by coded intent of injury. The percentage of hospitalizations for traumatic injury among young adults by injury intent across 12 states from January 2011 to December 2017. From top left to bottom right: Unintentional, Intentional Self-Harm, Assault, Undetermined

There was an immediate 1.1% level increase in the percentage of hospitalizations coded as resulting from unintentional injuries (*p* = 0.046), a 1.0% decrease in hospitalizations coded as resulting from assault (*p* = 0.007), and a 0.15% decrease in hospitalizations coded as resulting from injuries of undetermined intent (*p* = 0.02) in the first month after the coding transition (Fig. 2). In descriptive analyses, there were decreases in the percentages of assault injury hospitalizations for injury mechanisms of cut/pierce (− 0.5%) and unspecified mechanism (− 0.3%), which were mirrored by increases in the percentages of unintentional injury hospitalizations for the same two mechanisms (0.2 and 0.7%, respectively, results not shown) in October 2015. Removal of the October 2015 data point resulted in similar estimates for the immediate level changes in the percentages of hospitalizations coded for unintentional, assault, and undetermined injury intent (Table A1.5). Estimates of the immediate level changes in the percentages of hospitalizations coded for unintentional, assault, and undetermined injury intent were also similar when analyses were restricted to states with high ECOI completion. While there was no significant change in the trend in the proportion of hospitalizations coded as being for unintentional injuries in the main analysis, this proportion was found to increase at an average monthly rate of 0.06% after the coding transition among the states with high ECOI completion (*p* = 0.01 for the change in trend; Table A1.4). There was no immediate level change or change in trend after the coding transition in the percentage of hospitalizations coded as being for intentional self-harm injuries in either the main analysis or any sensitivity analyses.

### Injury severity

When ICDPIC version 0.1.0 was used, 51.6% of hospitalizations in the study population were classified as being for mild injury during the ICD-10-CM period, compared to 45.1% when using our updated version of ICDPIC-R. This difference was driven in part by the proportion of hospitalizations that received an ISS of 0 in the ICD-10-CM period, which was 6.5% when using ICDPIC-R version 0.1.0 and 1.8% when using the updated ICDPIC-R. Using ISS calculated from the updated ICDPIC-R software and the GEMmin method, the coding transition was associated with an immediate 4.0% increase in the percentage of hospitalizations classified as being for mild injury (*p* < 0.0001), a 4.3% decrease in the percentage of hospitalizations for moderate injury (*p* < 0.0001), and no change in the percentage of hospitalizations classified as being for severe injury. When ISS calculated from the updated ICDPIC-R and the GEMmax method were used, the estimated immediate level changes associated with the coding transition were a 1.2% increase in the percentage of hospitalizations for mild injury (*p* = 0.126), a 7.7% decrease in the percentage of moderate injury-related hospitalizations (*p* < 0.0001), and a 6.7% increase in the percentage of severe injury-related hospitalizations (*p* < 0.0001). In addition, there was a steeper positive trend over time in the percentage of hospitalizations for severe injury post-transition (0.17% average monthly increase after the transition; *p* = 0.002 for the change in trends) (Table 1). When ISS were calculated using ICDPIC-R version 0.1.0 and the GEMmin or GEMmax methods, the estimated immediate increases in percentages of hospitalizations for mild injury were larger (9.9 and 7.3%, *p* < 0.0001 for both), while the estimated changes in the percentages of hospitalizations for severe injury were similar to those obtained using our updated version of ICDPIC-R (Table 1). Results were similar to the main analyses, using either software version, after excluding the October 2015 data point and after restricting to states with high ECOI completion (Tables A1.4–5).

**Table 1:** Effects of transition to ICD-10-CM on observed injury severity, mechanism of injury, and traumatic brain injury-related hospitalization trends among young adults

	Initial level (Jan 2011)	Monthly trend before transition	Level change after transition	Trend change after transition
**Severity of Injury, % (ICDPIC-R Version 1)**^**a**^				
Mild, GEMmin (ISS: 0–8)	50.1	−0.12*	9.9*	0.01
Moderate, GEMmin (ISS: 9–15)	32.6	0.04‡	−9.2*	−0.04
Severe, GEMmin (ISS > 15)	17.6	0.07*	−0.4	0.04
Mild, GEMmax (ISS: 0–8)	50.0	−0.12*	7.3*	0.01
Moderate, GEMmax (ISS: 9–15)	32.5	0.05‡	−12.8*	−0.06
Severe, GEMmax (ISS > 15)	17.7	0.06*	6.0*	0.07‡
**Severity of Injury, % (Updated ICDPIC-R)**^**a, b**^				
Mild, GEMmin (ISS: 0–8)	50.0	−0.12*	4.0*	−0.03
Moderate, GEMmin (ISS: 9–15)	32.6	0.04‡	−4.3*	−0.02
Severe, GEMmin (ISS > 15)	17.6	0.07*	0.6	0.07‡
Mild, GEMmax (ISS: 0–8)	50.0	−0.12*	1.2	−0.03
Moderate, GEMmax (ISS: 9–15)	32.6	0.04‡	−7.7*	−0.05
Severe, GEMmax (ISS > 15)	17.5	0.07*	6.7*	0.10‡
**Mechanism of Injury, Rate per 100,000**^**c**^				
Cut/Pierce	1.28	−0.006*	−0.04	0.005†
Drowning	0.01	0.000	0.004†	0.000
Fall	3.52	−0.008*	−0.02	0.002
Fire/Burn	0.61	−0.001†	−0.23*	−0.001
Firearm	1.17	0.002	0.12‡	−0.001
Machinery	0.29	−0.002*	0.04†	0.001
MVT	5.50	−0.003	− 0.03	−0.007
Pedal Cyclist, Other	0.30	−0.001*	−0.05‡	−0.001
Pedestrian, Other	0.04	0.000	0.12*	−0.002‡
Transportation, Other	0.87	−0.004*	−0.11†	0.001
Natural/Environmental	0.16	−0.001*	−0.09*	0.000
Struck by/Against	1.54	−0.007*	−0.01	0.007†
Suffocation	0.01	0.000	0.07*	−0.001*
Other specified	0.38	−0.001*	−0.11*	0.001
Unspecified	0.54	−0.001‡	0.25*	−0.009*
**TBI, Rate per 100,000**^**d**^	4.54	−0.017*	0.60*	0.002
Skull Fracture	1.44	−0.002	− 0.04	−0.001
Other/Unspecified Fracture of Skull/Facial Bones	0.15	0.000	0.36*	−0.004*
Intracranial Injury	4.06	−0.016*	0.60*	0.004

*ISS* Injury Severity Score, *GEM* General equivalence mappings for linking ICD-9 to ICD-10 codes, *TBI* Traumatic brain injury

^a^ Excludes patients with any burn injury diagnosis

^b^ ICDPIC-R Updated to recognize ICD-10-CM codes with 7th characters of “B” or “C” and FY 2017, 2018 updates

^c^ Excludes patients without an ECOI code

^d^ Subcategories are not mutually exclusive

*, *p* < .0001; †, *p* < .05; ‡, *p* < .01

### Mechanism of injury

The transition to ICD-10-CM was associated with immediate level changes without changes in trends in the rates of hospitalization (per 100,000 persons) for injuries due to the following mechanisms: drowning (0.004; *p* = 0.034), fire/burn (− 0.23; *p* < 0.0001), firearm (0.12; *p* = 0.007), machinery (0.04; *p* = 0.048), other pedal cyclist (− 0.05; *p* = 0.002), other transportation (− 0.11; *p* = 0.022), natural/environmental (− 0.09; *p* < 0.0001), and other specified (− 0.11; *p* < 0.0001). The transition was also associated with immediate level increases, followed by decreasing trends in rates of hospitalization for injuries due to the following mechanisms: other pedestrian (0.12 immediate increase, *p* < 0.0001; 0.002 monthly decline, *p* = 0.0001 for the change in trends), suffocation (0.07 immediate increase and − 0.001 monthly decline; *p* < 0.0001 for both level and trend changes), and unspecified mechanism (0.25 immediate increase and − 0.010 monthly decline; *p* < 0.0001 for both level and trend changes) (Table 1). Although there was no immediate level change in the rate of hospitalization for cut/pierce injuries, there was a less steep decline in the rate of hospitalization for this type of injury after the transition (0.001 monthly decline after vs. 0.006 before transition; *p* = 0.045). Results were similar to the main analysis after excluding the October 2015 data point and after restricting the analyses to states with high ECOI completion, except there was no longer an immediate level increase in the rate of hospitalization for injuries caused by machinery (Tables A1.4–5).

### Traumatic brain injury

There was an immediate level increase in the overall rate of TBI-related hospitalizations (0.60 per 100,000 persons; *p* < 0.0001) without a change in the monthly trend after the transition (Table 1). The most common TBI subcategory throughout the study period was intracranial injury (89.7% of TBI-related hospitalizations), followed by skull fracture (33.5%) and other/unspecified fracture of skull or facial bones (6.1%), with two remaining subcategories (injury of optic chiasm/visual cortex; crushing injury of skull) combined accounting for < 1% of the TBI-related hospitalizations. In separate models by TBI subcategory, the transition to ICD-10-CM was associated with immediate level increases in the rates of hospitalizations coded as related to intracranial injury (0.60 per 100,000; *p* < 0.0001) and other/unspecified fracture of skull or facial bones (0.36 per 100,000; *p* < 0.0001), and no significant change in the rate of hospitalizations coded as related to skull fracture. The transition was also followed by a steepening of the declining trend in the rate of hospitalizations for other/unspecified fracture of skull (− 0.004 monthly decline; *p* < 0.0001 for the change in trend). When the October 2015 data point was removed from analysis, the immediate level increases in the rates of overall TBI-related, intracranial injury-related, and other/unspecified fracture of skull-related hospitalizations remained (Table A1.5). Results were also similar when the analyses were restricted to the states with consistently high ECOI completion (Table A1.4). In analyses stratified by state, the immediate level increase in the overall rate of TBI-related hospitalizations was significant in 5 of the 12 states, with state-level estimates ranging from a non-significant change of 0.09 per 100,000 in Rhode Island (*p* = 0.818) to 1.02 per 100,000 in Kentucky (*p* = 0.002) (Figures A2.3–4 and Table A2.2).

## Discussion

This study found that, across 12 geographically and demographically diverse U.S. states, the transition to ICD-10-CM coding had mostly small or negligible impacts on hospital discharge database-derived trends in population-level rates of traumatic injury-related hospitalizations by mechanism of injury. However, ECOI completeness decreased immediately after the coding transition by more than 3% and had yet to fully rebound by the end of 2017. In addition, the percentage of traumatic injury-related hospitalizations coded as resulting from unintentional injuries increased while the percentages of hospitalizations coded as resulting from assault-related injuries or injuries of undetermined intent decreased immediately after the coding transition, with the latter showing a steady downward trend post-transition as compared to an upward trend pre-transition. The rate of hospitalizations classified as TBI-related also increased immediately after the coding transition. Finally, we found the coding transition to be associated with marked changes in the distribution of traumatic injury-related hospitalizations by estimated injury severity score. However, the changes in this distribution differed according to whether ICD-10-CM codes that mapped to multiple ICD-9-CM codes were assigned to the ICD-9-CM code associated with greater or lesser injury severity. Both assignment strategies resulted in a shift in the distribution of patients away from the moderate injury category. When the greater severity ICD-9-CM codes were assigned, the shift in distribution resulted in a relative increase in the proportions of hospitalizations for severe injury. When the lower severity ICD-9-CM codes were assigned, there was an increase in the proportion of hospitalizations classified as resulting from mild injury but no observed change in the proportion of hospitalizations classified as resulting from severe injury. Many of the changes identified in this study were anticipated due to the increased effort required to code injuries using the ICD-10-CM system as well as changes in code specificity, coding structure, and coding guidelines. However, all changes in trends, whether expected or unexpected, are important for injury epidemiologists and health services researchers to consider when conducting analyses using administrative healthcare data that span the ICD-9-CM to ICD-10-CM coding transition.

The complexity of the ICD-10-CM coding system and the resultant increase in the time required to code similar cases using the ICD-10-CM versus ICD-9-CM system likely contributed to the observed decrease in ECOI completion. The transition to ICD-10-CM included a sixteen-fold increase in the number of injury diagnosis codes and the addition of roughly 6000 ECOI codes, creating a tradeoff between informativeness and efficiency (Fenton and Benigni 2014). A study by the Veteran’s Health Administration leading up to the transition evaluated a group of expert coders by calculating productivity (workload per hour) using both ICD-9-CM and ICD-10-CM in the ambulatory and inpatient settings over a seven month period. The results showed a decrease in productivity when using ICD-10-CM in both settings. When considering inpatient data, there was a sharp increase in the time necessary to complete patient records in the first month after the transition. This was followed by a steady improvement in efficiency each month, but a persistent gap remained at the end of the study period (Weems et al. 2015). In addition to the increased effort required for ICD-10-CM coding, the drop in ECOI completeness may be driven by the fact that ECOI coding is not necessary for reimbursement and is not mandated in all states (Centers for Disease Control and Prevention 2008). Furthermore, even in states in which it is mandated, the mandate is not always enforced. This suggests that ECOI coding may suffer as coders look for ways to minimize the decrease in productivity brought on by the longer time necessary to code using the ICD-10-CM system (Topaz et al. 2013). The expectation is that the long-term benefit of improved data quality will far outweigh the consequences of a transient disruption in code completion or accuracy. We did find that the positive trend in ECOI completion that was present prior to the transition remained unchanged afterwards, providing reassurance that ECOI completion should continue to approach pre-transition levels. In the study by Slavova et al. that examined the effect of the coding system transition on injury hospitalization trends in the state of Kentucky, a drop in ECOI completion in October 2015 was also found, but the drop was only 1.7% and was followed by a return to pre-transition levels 1 month later (Slavova et al. 2018). Across the 12 states in our study, there was an immediate drop in ECOI completion of 3.7%, with a more modest recovery of 0.7% from October to November 2015. In both studies, the trend in the rate of ECOI completion remained the same before and after the transition. While the drop and subsequent post-transition uptick in ECOI completion reported in the study by Slavova et al. differ substantially from those found in our multi-state study, the discrepancy may be partly explained by the fact that the study by Slavova et al. included hospitalizations of patients of any age and resulting from any injury mechanism, while our study was restricted to non-elective hospitalizations among young adults that met the NTDS definition (Slavova et al. 2018; The American College of Surgeons 2020). This discrepancy also highlights the fact that the impact of the coding transition on hospitalization trends can be expected to vary across states and populations.

Changes in the distribution of injury-related hospitalizations by coded injury intent after the coding transition were anticipated due to the ICD-10-CM guidelines instructing coders to classify all injuries of unknown or unspecified intent as unintentional (Injury Surveillance Workgroup 9 2016; Slavova et al. 2018). Only when physician documentation in the medical record specifies that the intent cannot be determined is the designation of “undetermined” to be used (Injury Surveillance Workgroup 9 2016). Consistent with the expected changes, we found an immediate increase in the percentage of hospitalizations coded as resulting from unintentional injuries, and both an immediate decrease and decreasing trend in the percentage of hospitalizations coded as resulting from injuries of undetermined intent. However, while there was a 1.1% increase and 0.15% decrease in unintentional and undetermined intent injury hospitalizations respectively, there was also a 1% decrease in assault-related injury hospitalizations. These findings suggest that in this population of young adult trauma patients, the increase in hospitalizations coded for unintentional injuries was driven primarily by a decrease in coding of injuries for assault. Additional descriptive analyses revealed two injury mechanisms that drove this shift in hospitalizations being coded for unintentional injury rather than assault: cut/pierce and unspecified mechanism. Decreases in assault injury hospitalizations coded for each of these two mechanisms were mirrored by increases in unintentional injury hospitalizations coded for the same mechanisms.

Using the final ECOI matrix published by the CDC at the end of 2019, (Hedegaard et al. 2019) we found several level changes in rates of traumatic injury-related hospitalization by coded mechanism of injury immediately after the coding transition, although subsequent trends were mostly unchanged. A review of some of the ICD-10-CM ECOI codes and the updates discussed by the authors of the final ECOI matrix report shows that many of our findings for mechanism of injury changes can be at least partly explained by differences in the structure and placement of specific codes in the ICD-10-CM versus ICD-9-CM matrices, in addition to the increased specificity and number of ICD-10-CM codes (Hedegaard et al. 2019). For example, the addition of ICD-10-CM code T75.1 (unspecified effects of drowning and nonfatal submersion), which did not have an equivalent in ICD-9-CM, may have contributed to the immediate increase in the rate of injury hospitalizations due to drowning. The decision to move codes for injuries due to firearm malfunction (W32.0, W33.00–W33.09, W34.00, and W34.09) from “unintentional, other specified” to “unintentional, firearm”, may have contributed to both the immediate increase in injury hospitalizations resulting from firearms and the decrease in hospitalizations caused by other specified injury mechanisms. The decision to place code X58 (exposure to other specified factors) in “unintentional, unspecified” coupled with the deactivation of code X59 from ICD-10-CM may have contributed to both the decreases in rates of injury hospitalizations due to natural/environmental and other specified mechanisms and the increase in rates of injury hospitalizations due to unspecified mechanisms. Other changes in code placement, inclusion, specificity or description likely contributed to the immediate decrease in the rates of injury hospitalizations due to other pedal cyclist mechanism and immediate increases in injury hospitalizations due to machinery, other transportation, and suffocation, although potential reasons for the decrease in the rate of injury hospitalizations due to fire/burn are less clear (Hedegaard et al. 2019).

Importantly, this is the first population-based study to evaluate changes post ICD-10-CM in estimates of injury severity based on diagnosis codes. The Injury Severity Score (ISS) was created to stratify patients based on the cumulative trauma present at the time of hospital presentation. Traditionally, calculation required patient observation or manual review of a patient’s medical record to determine the most severe injury to six separate body regions. However, the development of tools to convert ICD injury diagnosis codes to abbreviated injury scale (AIS) scores and ISS provided an effective method for stratifying large groups of patients without necessitating laborious review of the medical record (Tohira et al. 2012; Abajas-Bustillo et al. 2019). The large increase in the number of injury diagnosis codes from ICD-9-CM to ICD-10-CM has resulted in a one-to-many mapping of many ICD-9-CM diagnosis codes to ICD-10-CM diagnosis codes, making possible a range of ISS values after mapping. The GEM methods in ICDPIC-R are available to be used for data that span the coding transition, and these methods provide analysts the option to choose between a minimum calculated injury severity score (GEMmin) or maximum calculated injury severity score (GEMmax) for hospitalizations with ICD-10-CM injury diagnosis codes that map to multiple ICD-9-CM codes. No major updates of the initial ICDPIC-R version 0.1.0 have been made available in the public domain, and in the course of this study some important practical limitations of the initial version of this software were recognized. Specifically, ICDPIC-R version 0.1.0 requires ICD-10-CM codes in the input dataset to contain 7 characters, does not recognize codes with a 7th character other than “A”, and uses only the 2016 GEM (Issues. ICDPICR 2020). Our team revised and updated the ICDPIC-R software to recognize ICD-10-CM codes that are fewer than 7 digits, codes that end in “B” or “C” (relevant for open fracture codes), and codes that were added in the 2017 and 2018 fiscal year updates of the GEM. We found that use of ISS calculated from ICDPIC-R version 0.1.0 overestimated the effect of the coding transition on the percentage of mild injury hospitalizations compared to ISS derived from the updated ICDPIC-R, with immediate level increase estimates of 9.9% versus 4.0%, respectively, when the GEMmin method was used. In addition, while the GEMmax-derived estimate of the immediate level increase in mild injury hospitalizations was 7.3% using severity scores from ICDPIC-R version 0.1.0, this estimate was only 1.2% and no longer statistically significant after using the updated version of ICDPIC-R. The primary reason for the differences in effect estimates for the percentage of mild injury-related hospitalizations appears to be the percentage of hospitalizations assigned an ISS of 0 in the ICD-10-CM period, as this percentage was 6.5% when using ICDPIC-R version 0.1.0 and 1.8% when using the updated ICDPIC-R. Nevertheless, use of ISS derived from the GEMmax method, in either the initial or the updated version of ICDPIC-R, resulted in similar estimates of both the immediate level increase in the percentage of severe injury hospitalizations (6.0 and 6.7%, respectively) and the increase in the trend in severe injury hospitalizations post-transition. Patients with severe injuries are at greater risk of poor outcomes and are more likely to require intensive care services and discharge to an extended care facility (Gagné et al. 2017; Palmer et al. 2016). Therefore, the results of this study support the use of the GEMmin method over the GEMmax method in future studies using both ICD-9-CM and ICD-10-CM coded data that aim to either focus on severely injured patients or adjust for whether a patient is severely injured. We note, however, that the above-listed practical limitations of the initial version of ICDPIC-R can lead to overestimation of mild injuries in the ICD-10-CM period.

Lastly, we identified an immediate increase in the rate of hospitalizations coded as TBI-related after the transition to ICD-10-CM. TBI is a major cause of mortality and morbidity, and the financial and medical burdens of these injuries can be devastating (Najem et al. 2018). In order to further examine the effect of the coding transition separately by meaningful TBI subcategories, we used the TBI diagnosis groupings recommended in the ICD-10-CM TBI surveillance definition, and we identified the equivalent ICD-9-CM codes using the Barell injury diagnosis matrix and the 2017 GEMs (Hedegaard et al. 2016; Barell et al. 2002). We found that the immediate increase in the overall rate of hospitalizations coded as TBI-related after the coding transition was driven by immediate increases in the rates of hospitalizations with diagnosis codes that fall under the TBI subcategories of intracranial injury and other/unspecified fracture of skull or facial bones. In contrast, there were neither level nor trend changes in the rate of hospitalizations with diagnosis codes that fall under the TBI subcategory of skull fracture. A review of GEMs equivalent codes for intracranial injury suggests that some of the ICD-10-CM codes for traumatic cerebral edema (S06.1) map to codes that historically were not included in the ICD-9-CM Barell matrix TBI definition (e.g. 348.5, cerebral edema). Conversely, some of the codes for other specified fracture of skull or facial bones (S02.8), which are classified as TBI in the ICD-10-CM injury diagnosis matrix, map to ICD-9-CM codes that are classified elsewhere in the Barell matrix (e.g. 802, fracture of facial bones is placed in the “Other head, face, and neck” body region rather than classified as TBI in the Barell matrix). These findings suggest that although the ICD-10-CM surveillance definition for TBI was intended to align with the ICD-9-CM Barell matrix, some structural and conceptual changes affecting diagnosis codes for intracranial injury and other/unspecified fracture of skull or facial bones have led to an expanded definition of TBI in the ICD-10-CM injury diagnosis matrix. We also note that the number of codes available under S02.8 (other specified fracture of skull and facial bones) in particular has increased throughout updates of the ICD-10-CM, going from a single code (S02.8) in FY 2015 to the addition of separate codes indicating laterality (S02.80, S02.81, S02.82) in the 2017 update, and then the addition of separate codes for fractures of the orbital wall (S02.83, S02.84) and unspecified fracture of the orbit (S02.85) with the 2020 update. The most recently added codes S02.8[3–5], which do not describe TBIs, may result in a reduction in the number of hospitalizations classified as TBI-related starting in the year 2020, and thus may mitigate some of increase in TBI-related hospitalizations associated with the coding transition as reported in the present study. Researchers conducting TBI-related research studies using administrative healthcare data that spans the coding transition should consider stratifying or restricting analyses by TBI surveillance subcategories in order to control for these changes.

This study was not without limitations. Because encrypted patient identifiers were not available in all included states’ databases, we did not identify patients transferred between hospitals or readmitted. Thus, the data presented here reflect distinct hospitalizations rather than distinct injury events. Also, the combined maximum number of diagnosis and ECOI code fields available in the SID was constant across the years spanning the transition (2015–2016) in only 2 of the 12 states included in the study (Iowa, Rhode Island). Among the remaining states, 4 had an increase (Colorado, Georgia, Kansas, Nevada) and the remaining 6 had a decrease in the combined number of diagnosis and ECOI fields after the transition to ICD-10-CM. The changes in availability of diagnosis and ECOI fields after the transition could have impacted the findings for any of the outcomes in our study. However, it is difficult to estimate the magnitude and direction of such impacts given that the direction of the changes in numbers of diagnosis and ECOI code fields varied by state. Also, although the 12 states selected for this study together have a large and sociodemographically diverse young adult population, our study is not necessarily nationally representative. Because there is heterogeneity in the quality of coding and particularly ECOI coding across states, the trends we have identified may not reflect those seen in some particular states. Nevertheless, these results can be used to inform future research studies and coding quality improvement efforts.

## Conclusion

The transition to ICD-10-CM has resulted in disruptions in some observed trends in traumatic injury-related hospitalizations among young adults in the U.S.. The ICD-10-CM coding system’s complexity has required adjustments by both clinicians and coders, and these have resulted in a decrease in ECOI coding, though data quality remains high. Trends in rates of injury-related hospitalizations for several mechanisms of injury were impacted, in mostly expected ways, by the coding transition. Trends in rates of TBI-related hospitalizations and the distribution of injury-related hospitalizations by severity of injury were also impacted. It is essential that future injury-related research studies using administrative healthcare databases be designed with an awareness of these impacts, so that the changes do not inappropriately impact the interpretation of study findings. Future studies should also evaluate the impact of the coding transition on trends in the characteristics of injuries treated in outpatient settings and in the distribution of injuries by body region and nature of injury.

## Supplementary Information

**Additional file 1: Table A1.1.** Study inclusion criteria using injury diagnosis codes in the principal diagnosis field. **Table A1.2.** International Classification of Disease (ICD) Codes Used to Classify Traumatic Brain Injury (TBI) Admissions in the Study Population. **Table A1.3.** Number of included admissions by state. **Table A1.4.** Effects of the transition to ICD-10-CM on outcomes in states with >90% ECOI completion. **Table A1.5.** Segmented Regression Results After Removing October 2015 Data Point.

**Additional file 2: **ECOI Completion and TBI Rates by State. **Figure A2.1.** Effects of the transition to ICD-10-CM on ECOI in the study population, by state (AR-KY). **Figure A2.2.** Effects of the transition to ICD-10-CM on ECOI in the study population, by state (NC-RI). **Table A2.1.** Effects of the transition to ICD-10-CM on ECOI in the study population, by state. **Figure A2.3.** Effects of the transition to ICD-10-CM on TBI-related hospitalization rates in the study population, by state (AR-KY). **Figure A2.4.** Effects of the transition to ICD-10-CM on TBI-related hospitalization rates in the study population, by state (NC-RI). **Table A2.2.** Effects of the transition to ICD-10-CM on TBI-related hospitalization rates in the study population, by state.

## Data Availability

The HCUP data used in this study are available for purchase from the HCUP central distributor. Per the specifications of the HCUP state database data use agreement, the data cannot be shared by the authors. The authors declare that all aggregate data supporting the findings in this study are available within the article and supplementary files.

## Abbreviations

AIS: Abbreviated Injury Score
CDC: Centers for Disease Control and Prevention
ECOI: External Cause of Injury
HCUP: Healthcare Cost and Utilization Project
HIPPA: Health Insurance Portability and Accountability Act
ICD-CM: International Classification of Diseases, Clinical Modification
ISS: Injury Severity Score
NTDS: National Trauma Data Standards
TBI: Traumatic Brain Injury
