# Supplementary material for: Impact of ICD-9-CM to ICD-10-CM coding transition on trauma hospitalization trends among young adults in 12 states
Source: Inj Epidemiol. 2021 Jan 25;8:4. doi: 10.1186/s40621-021-00298-x (PMC7830822; doi:10.1186/s40621-021-00298-x)
Supplement: Supplementary file 1 — Additional file 1: Table A1.1. Study inclusion criteria using injury diagnosis codes in the principal diagnosis field. Table A1.2. International Classification of Disease (ICD) Codes Used to Classify Traumatic Brain Injury (TBI) Admissions in the Study Population. Table A1.3. Number of included admissions by state. Table A1.4. Effects of the transition to ICD-10-CM on outcomes in states with >90% ECOI completion. Table A1.5. Segmented Regression Results After Removing October 2015 Data Point. [file 40621_2021_298_MOESM1_ESM.docx]

**Additional file 1.** Supplemental Tables and Sensitivity Analyses

**Table A1.1** Study inclusion criteria using injury diagnosis codes in the principal diagnosis field

| ICD-9 CM  (for discharges prior to October 1, 2015) | | ICD-10-CM (for discharges on or after October 1, 2015) ^a^ | |
| --- | --- | --- | --- |
| Included | Excluded | Included | Excluded |
| 800-959 | 905-909: late effects of injury  910-924 (superficial injuries)  930-939 (foreign bodies)^b^ | S00-S99 (anatomic injuries; with 7^th^ character modifier A-R)  T07 (unspecified multiple injuries; with 7^th^ character modifier A, D)  T14 (injury of unspecified body region; with 7^th^ character modifier A, D)  T20-T28 (burns by specific body parts; with 7^th^ character modifier A, D)  T30-T32 (burn by TBSA percentages)  T79.A1-T79.A9 (Traumatic Compartment Syndrome; with 7^th^ character modifier A,D) | S10, S20, S30, S40, S50, S60, S70, S80, S90 (superficial injuries)  Any of the inclusion codes with 7^th^ character modifier of S (sequalae of injury) |
| *Notes.*  ^a^ Difference from the National Trauma Data Standard (NTDS) ICD-10-CM inclusion criteria: among the injury diagnosis codes listed for inclusion, codes that have a 7^th^ digit modifier code of D through R (subsequent encounters) are excluded from NTDS. All of the above-listed codes were selected for inclusion in the study population regardless of whether they are for a first or a subsequent encounter. The concepts of initial and subsequent encounter were not included in ICD-9-CM. Therefore, exclusion of cases based on having a 7^th^ digit code modifier for subsequent encounter could lead to a greater proportion of cases excluded in the ICD-10-CM era. However, because “subsequent encounters” are defined in ICD-10-CM as encounters for routine injury-related care during the healing or recovery process, we identified very few non-elective “subsequent” traumatic injury hospitalizations.  ^b^ Foreign body-related injuries are also excluded during the ICD-10-CM period as they are not among the list of included principal diagnosis codes (T15.xxx-T19.xxx) | | | |

**Table A1.2** International Classification of Disease (ICD) Codes Used to Classify Traumatic Brain Injury (TBI) Admissions in the Study Population

| TBI Subcategory | ICD–10–CM^a^ | ICD-9-CM^b^ |
| --- | --- | --- |
| Skull Fracture | S02.0, S02.1 | 800-801 |
| Other/Unspecified Fracture of Skull/Facial Bones | S02.8, S02.91 | 803-804 |
| Injury of optic chiasm/optic tract and pathways/visual cortex | S04.02, S04.03, S04.04 | 950(.1-.3) |
| Intracranial injury | S06 | 850-854, 995.55, [800, 801, 803, 804] (.10-.49; .60-.99) |
| Crushing injury of skull | S07.1 | General Equivalence Mapping equivalent not classified as TBI in Barell Matrix |
| ^a^ ICD-10 codes obtained from Table C in Hedegaard H, Johnson RL, Warner M, Chen L-H, Annest JL. Proposed Framework for Presenting Injury Data Using the International Classification of Diseases, Tenth Revision, Clinical Modification Diagnosis Codes. National Health Statistics Reports; no 89. Hyattsville, MD: National Center for Health Statistics; 2016. | | |
| ^b^ ICD-9 Codes adapted from Barell V, Aharonson-Daniel L, Fingerhut LA, Mackenzie EJ, Ziv A, Boyko V, et al. An introduction to the Barell body region by nature of injury diagnosis matrix. Inj Prev. 2002;8(2):91-6. | | |

**Table A1.3.** Number of included admissions by state

| **Year** | **2011** | **2012** | **2013** | **2014** | **2015** | **2016** | **2017** | **Total** |
| --- | --- | --- | --- | --- | --- | --- | --- | --- |
| Arkansas | 2217 | 2338 | 2018 | 2060 | 2082 | 2120 | 1961 | 14796 |
| Colorado | 4279 | 4257 | 4065 | 3851 | 4122 | 4259 | 4192 | 29025 |
| Georgia | 7347 | 7237 | 7374 | 6908 | 7383 | 7841 | 7427 | 51517 |
| Iowa | 1866 | 1851 | 1640 | 1497 | 1548 | 1640 | 1729 | 11771 |
| Kansas | 2116 | 2082 | 1777 | 1715 | 1831 | 1850 | 1858 | 13229 |
| Kentucky | 3014 | 3028 | 2793 | 2738 | 2815 | 2883 | 2835 | 20106 |
| North Carolina | 7158 | 6775 | 6225 | 5927 | 6271 | 6620 | 6452 | 45428 |
| New Jersey | 7122 | 6749 | 6466 | 5958 | 5973 | 5508 | 4860 | 42636 |
| New Mexico | 1571 | 1416 | 1373 | 1379 | 1257 | 1387 | 1383 | 9766 |
| Nevada | 1984 | 2040 | 2033 | 2036 | 2127 | 2173 | 2171 | 14564 |
| Oregon | 2609 | 2627 | 2420 | 2194 | 2425 | 2555 | 2417 | 17247 |
| Rhode Island | 726 | 666 | 592 | 581 | 589 | 606 | 594 | 4354 |
| Total | 42009 | 41066 | 38776 | 36844 | 38423 | 39442 | 37879 | 274439 |

**Table A1.4.** Effects of the transition to ICD-10-CM on outcomes in states with >90% ECOI completion

|  | **Initial level (Jan 2011)** | **Monthly trend before transition** |  | **Level change after transition** |  | **Trend change after  transition** |  |
| --- | --- | --- | --- | --- | --- | --- | --- |
| ECOI Completion, %^a^ | 96.6 | 0.01 |  | -1.9 | * | 0.02 |  |
| **Intent of Injury, %^b^** |  |  |  |  |  |  |  |
| Unintentional | 80.4 | 0.00 |  | 0.95 | † | 0.06 | ‡ |
| Intentional Self-Harm | 2.1 | 0.01 | ‡ | 0.22 |  | -0.01 |  |
| Assault | 16.6 | -0.01 |  | -0.96 | † | -0.05 |  |
| Undetermined | 0.59 | 0.003 | ‡ | -0.18 | † | -0.007 |  |
| **Severity of Injury, % (ICDPIC-R Version 1) ^c^** |  |  |  |  |  |  |  |
| Mild, GEMmin (ISS: 0-8) | 49.9 | -0.10 | * | 9.7 | * | -0.050 |  |
| Moderate, GEMmin (ISS: 9-15) | 32.8 | 0.04 | † | -9.2 | * | -0.015 |  |
| Severe, GEMmin (ISS>15) | 17.5 | 0.06 | * | -0.4 |  | 0.057 | † |
| Mild, GEMmax (ISS: 0-8) | 49.9 | -0.10 | * | 7.2 | * | -0.053 |  |
| Moderate, GEMmax (ISS: 9-15) | 32.6 | 0.04 | † | -13.0 | * | -0.040 |  |
| Severe, GEMmax (ISS>15) | 17.5 | 0.06 | * | 6.0 | * | 0.078 | ‡ |
| **Severity of Injury, % (Updated ICDPIC-R) ^c,d^** |  |  |  |  |  |  |  |
| Mild, GEMmin (ISS: 0-8) | 49.9 | -0.11 | * | 3.9 | * | -0.057 |  |
| Moderate, GEMmin (ISS: 9-15) | 32.8 | 0.04 | † | -4.1 | * | -0.016 |  |
| Severe, GEMmin (ISS>15) | 17.5 | 0.06 | * | 0.7 |  | 0.085 | ‡ |
| Mild, GEMmax (ISS: 0-8) | 50.0 | -0.11 | * | 1.1 |  | -0.057 |  |
| Moderate, GEMmax (ISS: 9-15) | 32.7 | 0.04 | † | -7.6 | * | -0.042 |  |
| Severe, GEMmax (ISS>15) | 17.5 | 0.06 | * | 7.0 | * | 0.105 | * |
| **Mechanism of Injury, Rate per 100,000^b^** |  |  |  |  |  |  |  |
| Cut/Pierce | 1.043 | -0.005 | * | 0.01 |  | 0.003 |  |
| Drowning | 0.006 | 0.000 |  | 0.00 |  | 0.000 | † |
| Fall | 2.968 | -0.008 | * | 0.02 |  | 0.003 |  |
| Fire/Burn | 0.548 | -0.001 | ‡ | -0.20 | * | 0.000 |  |
| Firearm | 1.022 | 0.001 |  | 0.13 | ‡ | -0.002 |  |
| Machinery | 0.257 | -0.002 | * | 0.02 |  | 0.002 |  |
| MVT | 4.792 | -0.008 | † | 0.08 |  | -0.003 |  |
| Pedal Cyclist, Other | 0.265 | -0.001 | * | -0.04 | † | -0.001 |  |
| Pedestrian, Other | 0.035 | 0.000 |  | 0.10 | * | -0.002 | * |
| Transportation, Other | 0.717 | -0.003 | * | -0.09 | † | 0.002 |  |
| Natural/Environmental | 0.126 | -0.0004 | ‡ | -0.07 | * | 0.0002 |  |
| Struck by/Against | 1.314 | -0.007 | * | 0.02 |  | 0.005 |  |
| Suffocation | 0.013 | 0.000 |  | 0.05 | * | -0.001 | ‡ |
| Other specified | 0.314 | -0.001 | * | -0.08 | * | 0.001 |  |
| Unspecified | 0.458 | -0.001 | † | 0.18 | * | -0.007 | * |
| **TBI, Rate per 100,000^e^** | 3.84 | -0.015 | * | 0.44 | * | 0.005 |  |
| Skull Fracture | 1.18 | -0.002 | † | -0.01 |  | 0.000 |  |
| Other/Unspecified Fracture of Skull/Facial Bones | 0.13 | 0.000 |  | 0.29 | * | -0.004 | ‡ |
| Intracranial Injury | 3.42 | -0.013 | * | 0.38 | ‡ | 0.007 |  |
| *, p<.0001; †, p<.05; ‡, p<.01; | | | | | | | |
| ISS, Injury Severity Score; GEM, general equivalence mappings for linking ICD-9 to ICD-10 codes | | | | | | | |
| ^a^ Excludes December 2017 data. | | | | | | | |
| ^b^ Excludes patients without an ECOI code | | | | | | | |
| ^c^ Excludes patients with any burn diagnosis | | | | | | | |
| ^d^ ICDPIC-R Updated to recognize ICD-10-CM codes with 7th characters of “B”/”C” and FY 2017, 2018 updates | | | | | | | |
| ^e^ Subcategories are not mutually exclusive | | | | | | | |

**Table A1.5.** Segmented Regression Results After Removing October 2015 Data Point

|  | **Initial level (Jan 2011)** | **Monthly trend before transition** |  | **Level change after transition** |  | **Trend change after  transition** |  |
| --- | --- | --- | --- | --- | --- | --- | --- |
| **Intent of Injury, %^a^** |  |  |  |  |  |  |  |
| Unintentional | 79.85 | 0.01 |  | 1.2 | † | 0.03 |  |
| Intentional Self-Harm | 2.31 | 0.01 | ‡ | 0.1 |  | 0.00 |  |
| Assault | 16.88 | -0.02 | † | -1.1 | ‡ | -0.02 |  |
| Undetermined | 0.58 | 0.003 | ‡ | -0.1 | † | -0.01 | † |
| **Severity of Injury, % (ICDPIC-R Version 1)^b^** |  |  |  |  |  |  |  |
| Mild, GEMmin (ISS: 0-8) | 50.11 | -0.119 | * | 9.5 | * | 0.032 |  |
| Moderate, GEMmin (ISS: 9-15) | 32.57 | 0.042 | ‡ | -8.8 | * | -0.054 |  |
| Severe, GEMmin (ISS>15) | 17.58 | 0.066 | * | -0.4 |  | 0.040 |  |
| Mild, GEMmax (ISS: 0-8) | 50.12 | -0.119 | * | 7.0 | * | 0.026 |  |
| Moderate, GEMmax (ISS: 9-15) | 32.49 | 0.044 | ‡ | -12.6 | * | -0.074 |  |
| Severe, GEMmax (ISS>15) | 17.68 | 0.063 | * | 6.2 | * | 0.059 | † |
| **Severity of Injury, % (Updated ICDPIC-R)^b,c^** |  |  |  |  |  |  |  |
| Mild, GEMmin (ISS: 0-8) | 50.10 | -0.119 | * | 3.7 | * | -0.009 |  |
| Moderate, GEMmin (ISS: 9-15) | 32.60 | 0.041 | ‡ | -4.0 | * | -0.039 |  |
| Severe, GEMmin (ISS>15) | 17.58 | 0.066 | * | 0.8 |  | 0.065 | ‡ |
| Mild, GEMmax (ISS: 0-8) | 50.10 | -0.119 | * | 1.0 |  | -0.012 |  |
| Moderate, GEMmax (ISS: 9-15) | 32.56 | 0.043 | ‡ | -7.6 | * | -0.060 |  |
| Severe, GEMmax (ISS>15) | 17.52 | 0.069 | * | 6.9 | * | 0.089 | ‡ |
| **Mechanism of Injury, Rate per 100,000^d^** |  |  |  |  |  |  |  |
| Cut/Pierce | 1.285 | -0.006 | * | -0.039 |  | 0.005 | † |
| Drowning | 0.008 | 0.000 |  | 0.004 | † | 0.000 |  |
| Fall | 3.518 | -0.008 | * | -0.030 |  | 0.003 |  |
| Fire/Burn | 0.606 | -0.001 | ‡ | -0.223 | * | -0.002 |  |
| Firearm | 1.170 | 0.002 |  | 0.112 | † | 0.000 |  |
| Machinery | 0.289 | -0.002 | * | 0.030 |  | 0.002 |  |
| MVT | 5.480 | -0.002 |  | 0.024 |  | -0.010 |  |
| Pedal Cyclist, Other | 0.301 | -0.001 | * | -0.053 | ‡ | -0.001 |  |
| Pedestrian, Other | 0.043 | 0.000 |  | 0.128 | * | -0.003 | * |
| Transportation, Other | 0.873 | -0.004 | * | -0.114 | † | 0.002 |  |
| Natural/Environmental | 0.161 | -0.001 | * | -0.086 | * | 0.000 |  |
| Struck by/Against | 1.573 | -0.008 | * | 0.027 |  | 0.008 | ‡ |
| Suffocation | 0.013 | 0.000 |  | 0.061 | * | -0.001 | ‡ |
| Other specified | 0.376 | -0.001 | ‡ | -0.100 | * | 0.001 |  |
| Unspecified | 0.539 | -0.001 | ‡ | 0.247 | * | -0.009 | * |
| **TBI, Rate per 100,000^e^** | 4.55 | -0.018 | * | 0.63 | * | 0.002 |  |
| Skull Fracture | 1.44 | -0.002 |  | -0.03 |  | -0.002 |  |
| Other/Unspecified Fracture of Skull/Facial Bones | 0.15 | 0.000 |  | 0.35 | * | -0.004 | ‡ |
| Intracranial Injury | 4.06 | -0.016 | * | 0.60 | * | 0.004 |  |
| *, p<.0001; †, p<.05; ‡, p<.01; | | | | | | | |
| ISS, Injury Severity Score; GEM, general equivalence mappings for linking ICD-9 to ICD-10 codes | | | | | | | |
| ^a^ Excludes patients without an ECOI code | | | | | | | |
| ^b^ Excludes patients with any burn diagnosis | | | | | | | |
| ^c^ ICDPIC-R Updated to recognize ICD-10-CM codes with 7th characters of “B”/”C” and FY 2017, 2018 updates | | | | | | | |
| ^d^ Subcategories are not mutually exclusive | | | | | | | |
