# Supplementary material for: Impact of ICD-9-CM to ICD-10-CM coding transition on trauma hospitalization trends among young adults in 12 states
Source: Inj Epidemiol. 2021 Jan 25;8:4. doi: 10.1186/s40621-021-00298-x (PMC7830822; doi:10.1186/s40621-021-00298-x)
Supplement: Supplementary file 2 — Additional file 2: ECOI Completion and TBI Rates by State. Figure A2.1. Effects of the transition to ICD-10-CM on ECOI in the study population, by state (AR-KY). Figure A2.2. Effects of the transition to ICD-10-CM on ECOI in the study population, by state (NC-RI). Table A2.1. Effects of the transition to ICD-10-CM on ECOI in the study population, by state. Figure A2.3. Effects of the transition to ICD-10-CM on TBI-related hospitalization rates in the study population, by state (AR-KY). Figure A2.4. Effects of the transition to ICD-10-CM on TBI-related hospitalization rates in the study population, by state (NC-RI). Table A2.2. Effects of the transition to ICD-10-CM on TBI-related hospitalization rates in the study population, by state. [file 40621_2021_298_MOESM2_ESM.docx]

**Additional file 2. ECOI Completion and TBI Rates by State**

**Figure A2.1** Effects of the transition to ICD-10-CM on ECOI in the study population, by state (AR-KY).


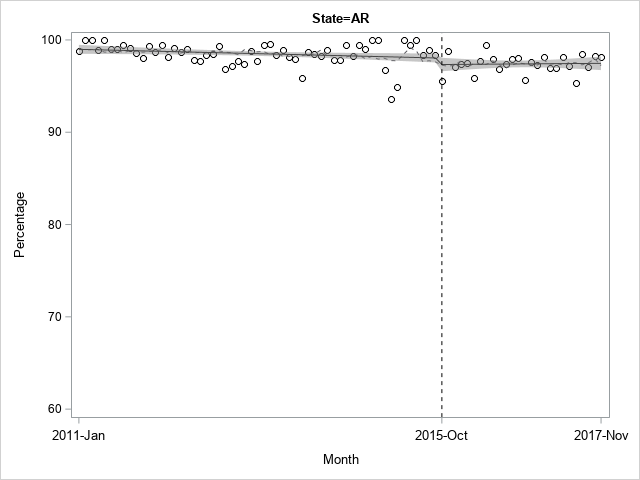

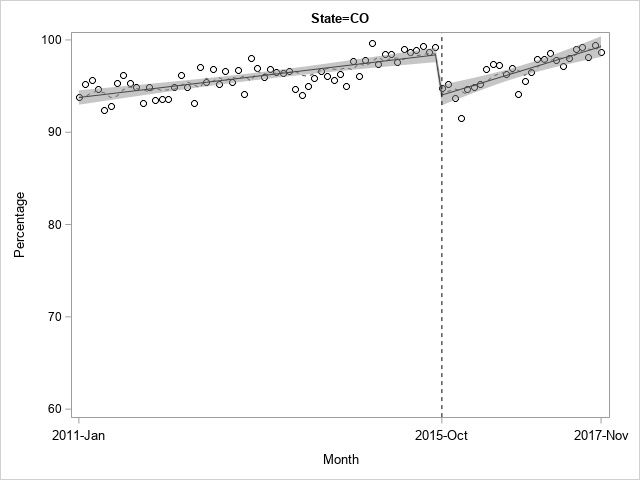

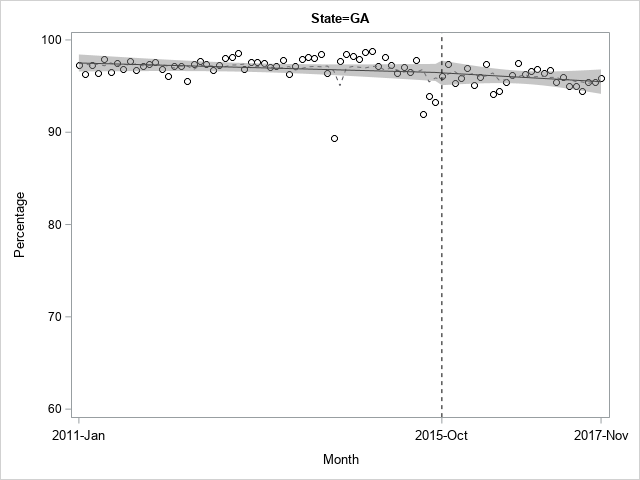

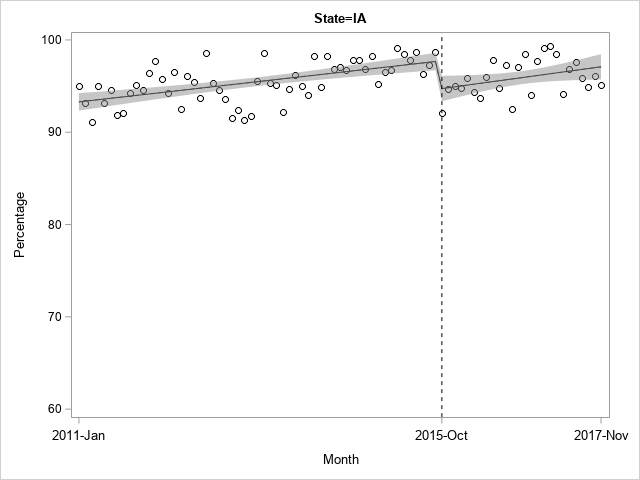

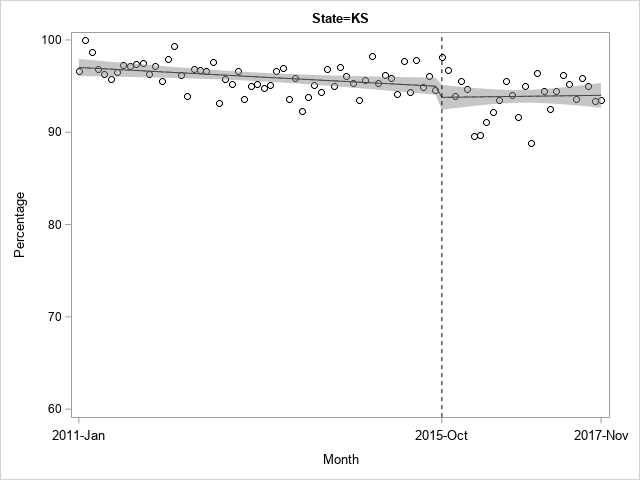

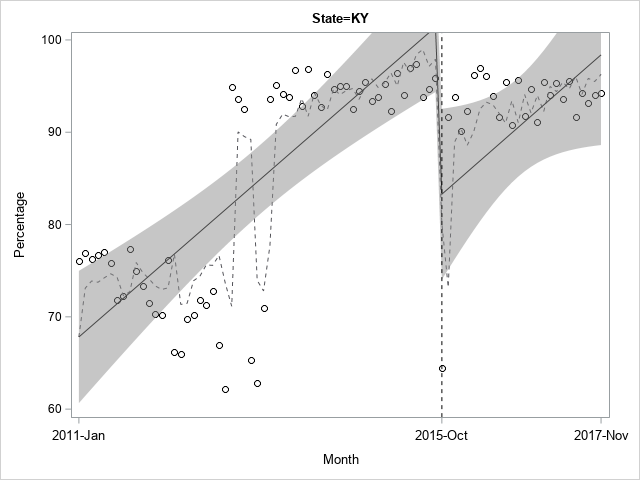


**Figure A2.2** Effects of the transition to ICD-10-CM on ECOI in the study population, by state (NC-RI).


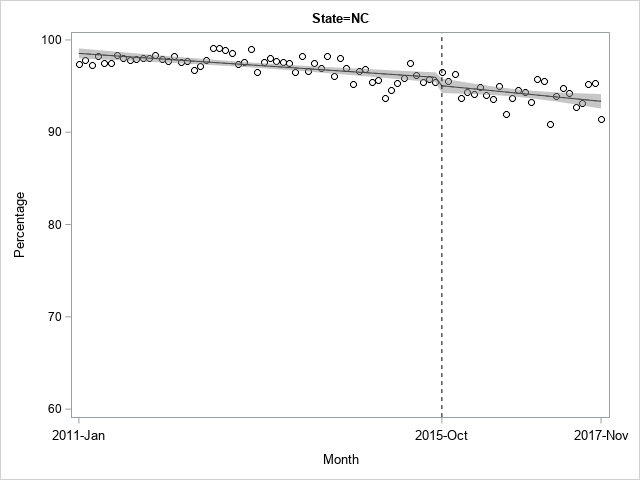

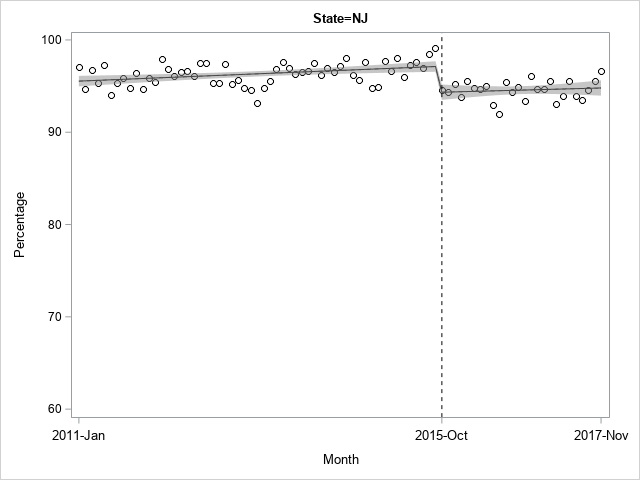

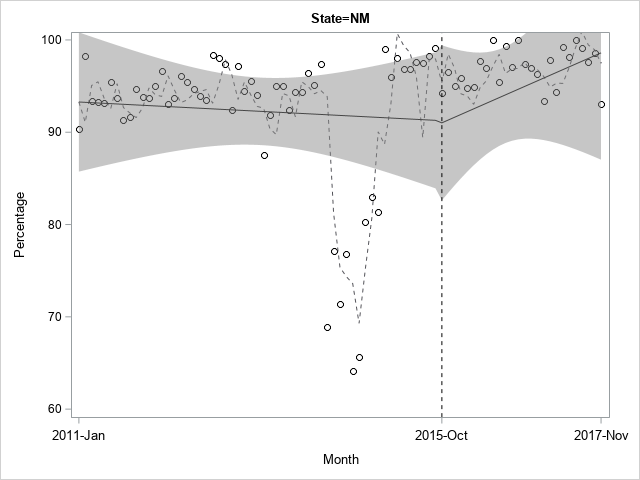

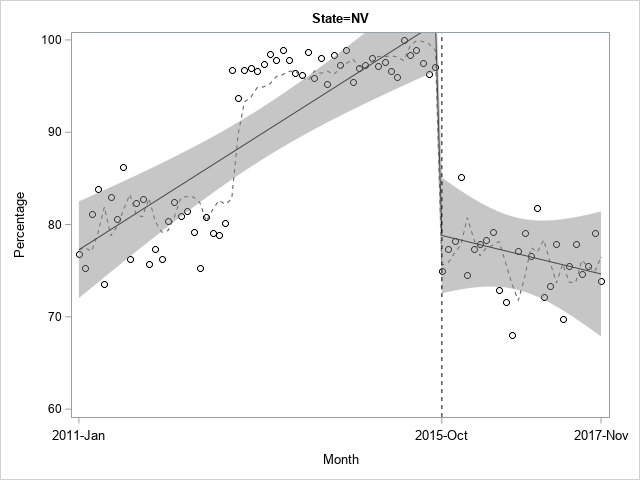

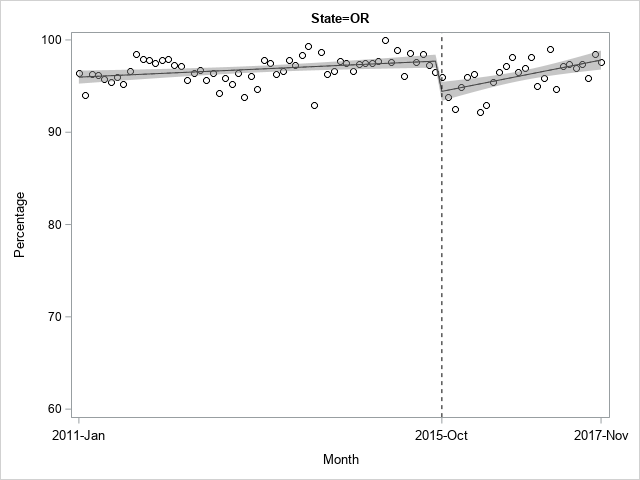

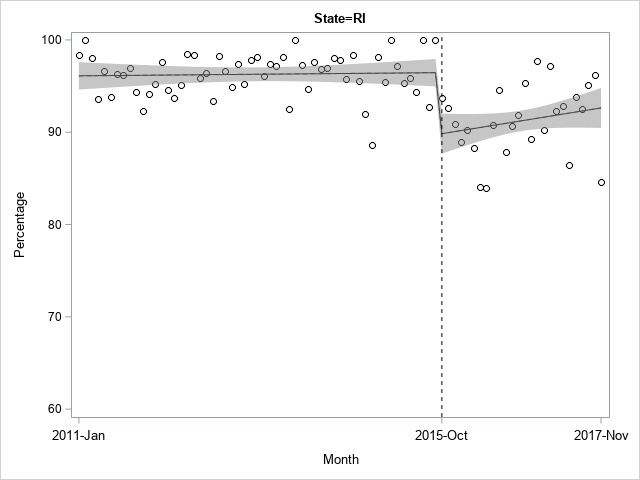


**Table A2.1.** Effects of the transition to ICD-10-CM on ECOI in the study population, by state

| State | Initial level (Jan 2011) | Monthly trend before transition | | Level change after transition | | Trend change after  transition | | >90% ECOI throughout | State  Mandate for ECOI codes |
| --- | --- | --- | --- | --- | --- | --- | --- | --- | --- |
| Arkansas | 99.0 | -0.02 | † | -0.7 |  | 0.02 |  | Yes | Yes |
| Colorado | 93.7 | 0.08 | * | -4.6 | * | 0.13 | ‡ | Yes | No |
| Georgia | 97.5 | -0.02 |  | 0.0 |  | -0.02 |  | Yes | Yes |
| Iowa | 93.2 | 0.08 | * | -3.0 | ‡ | 0.02 |  | Yes | No |
| Kansas | 97.1 | -0.04 | † | -1.2 |  | 0.04 |  | Yes | No |
| Kentucky | 67.2 | 0.60 | * | -18.7 | ‡ | 0.00 |  | No | Yes |
| North Carolina | 98.6 | -0.05 | * | -0.8 |  | -0.02 |  | Yes | No |
| New Jersey | 95.5 | 0.03 | ‡ | -2.8 | * | -0.01 |  | Yes | Yes |
| New Mexico | 93.3 | -0.04 |  | -0.6 |  | 0.34 |  | No | No |
| Nevada | 76.8 | 0.44 | * | -22.8 | * | -0.60 | † | No | Yes |
| Oregon | 95.9 | 0.03 | ‡ | -3.4 | * | 0.10 | ‡ | Yes | Yes |
| Rhode Island | 96.1 | 0.01 |  | -6.7 | * | 0.11 |  | No | Yes |
| Excludes December 2017 data. | | | | | | | | | |
| *, p<.0001; †, p<.05; ‡, p<.01; | | | | | | | | | |

**Figure A2.3.** Effects of the transition to ICD-10-CM on TBI-related hospitalization rates in the study population, by state (AR-KY).


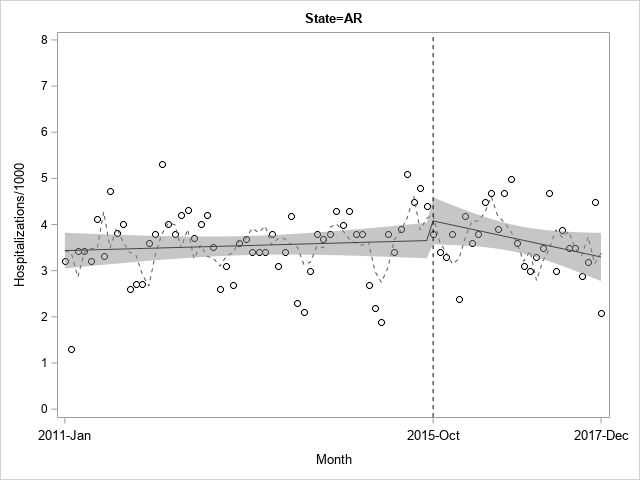

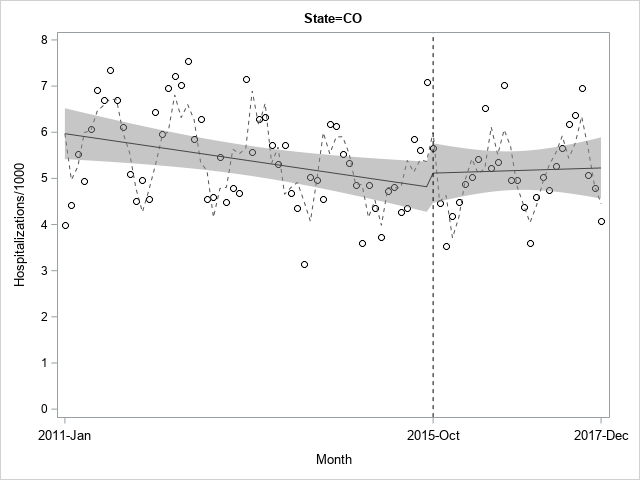

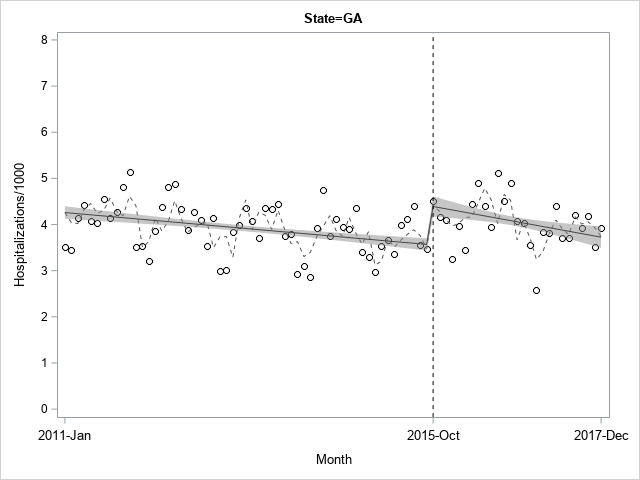

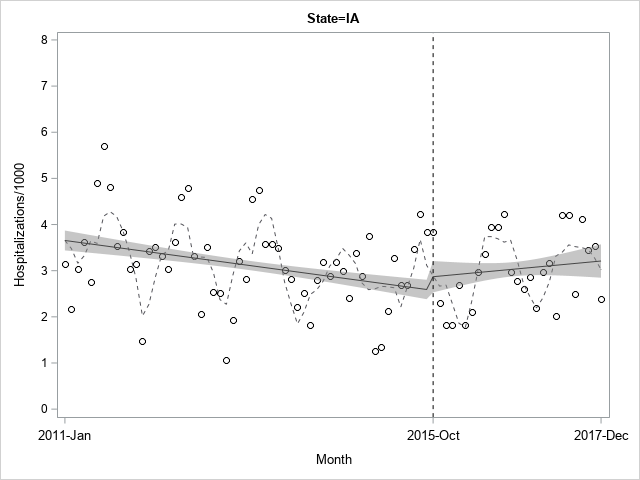

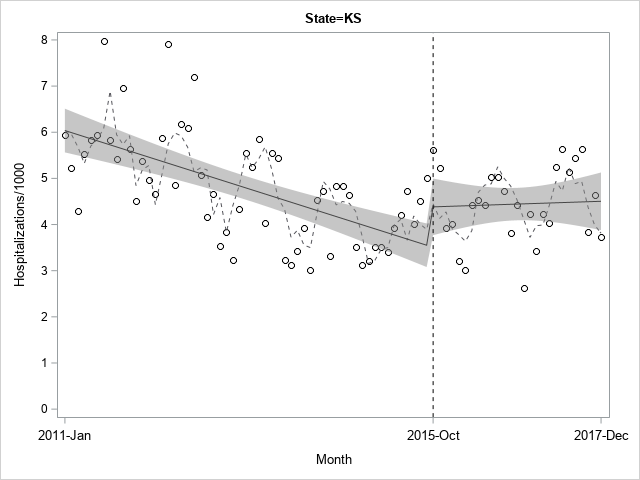

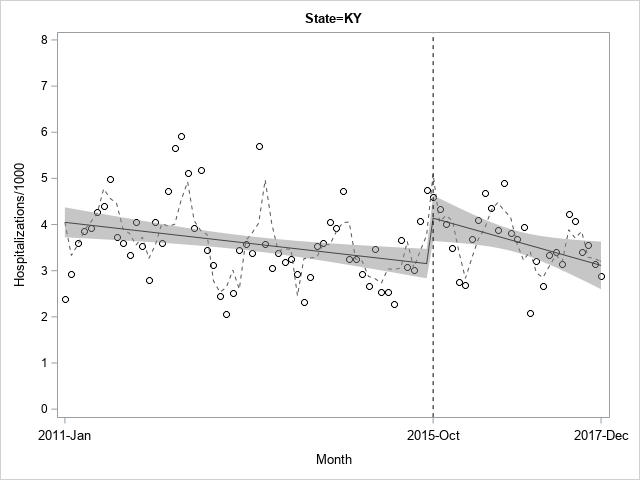


**Figure A2.4.** Effects of the transition to ICD-10-CM on TBI-related hospitalization rates in the study population, by state (NC-RI).


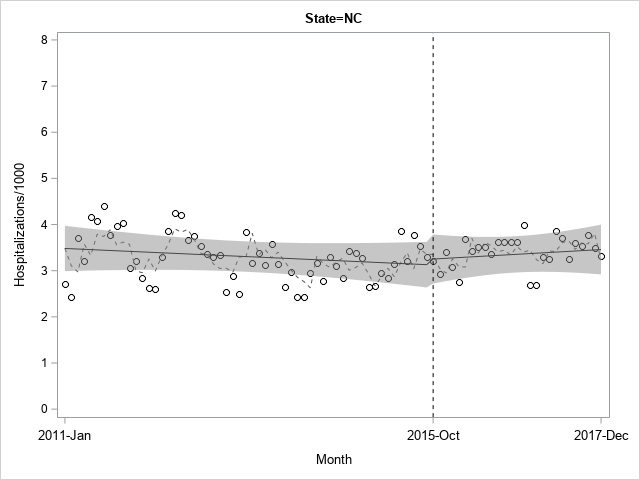

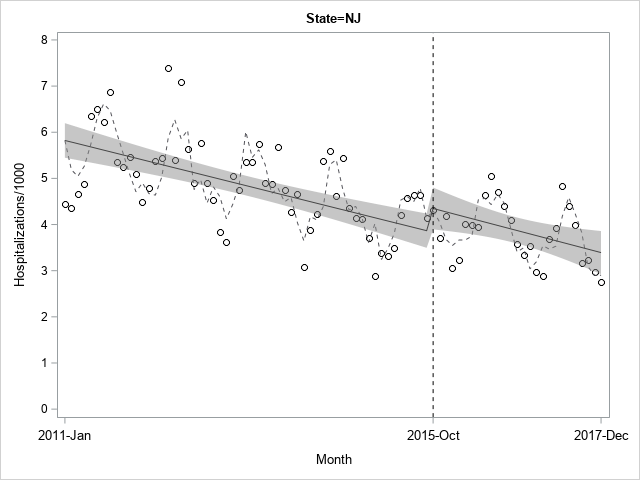

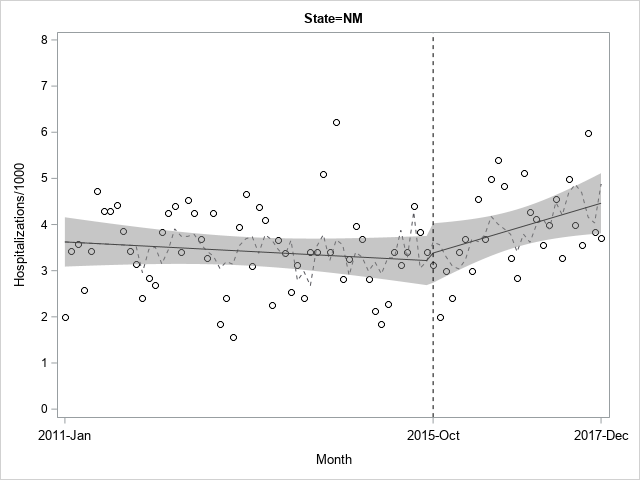

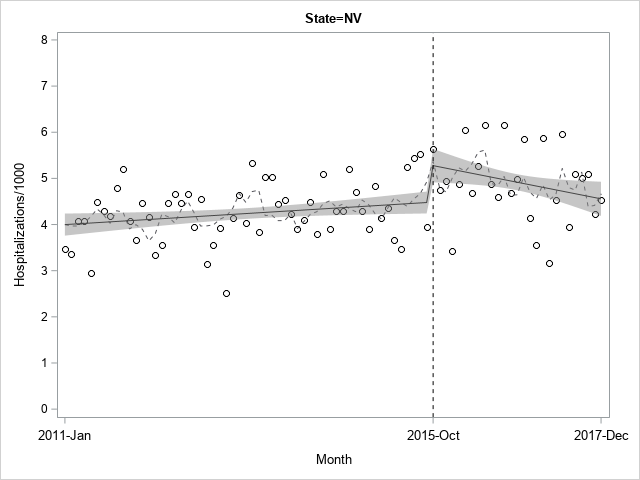

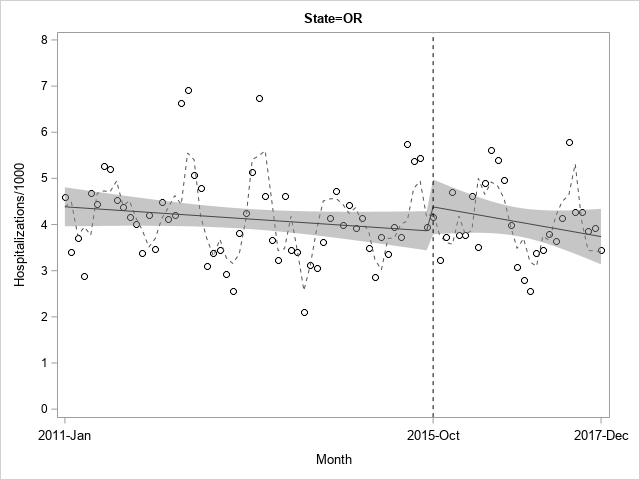

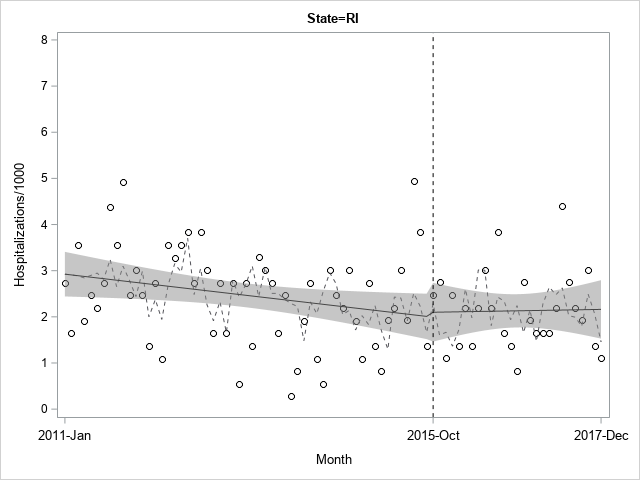


**Table A2.2.** Effects of the transition to ICD-10-CM on TBI-related hospitalization rates in the study population, by state

| State | Initial level (Jan 2011) | Monthly trend before transition | | Level change after transition | | Trend change after  transition | |
| --- | --- | --- | --- | --- | --- | --- | --- |
| Arkansas | 3.43 | 0.004 |  | 0.46 |  | -0.034 |  |
| Colorado | 5.99 | -0.021 | † | 0.29 |  | 0.025 |  |
| Georgia | 4.27 | -0.012 | * | 0.85 | * | -0.013 |  |
| Iowa | 3.67 | -0.019 | * | 0.26 |  | 0.032 | † |
| Kansas | 6.08 | -0.044 | * | 0.83 | † | 0.049 | † |
| Kentucky | 4.06 | -0.016 | ‡ | 1.02 | ‡ | -0.023 |  |
| North Carolina | 3.49 | -0.006 |  | 0.11 |  | 0.014 |  |
| New Jersey | 5.86 | -0.035 | * | 0.52 | † | -0.002 |  |
| New Mexico | 3.63 | -0.007 |  | 0.12 |  | 0.049 | † |
| Nevada | 3.99 | 0.009 | † | 0.83 | ‡ | -0.036 | ‡ |
| Oregon | 4.39 | -0.009 |  | 0.54 |  | -0.015 |  |
| Rhode Island | 2.94 | -0.016 |  | 0.09 |  | 0.019 |  |
| *, p<.0001; †, p<.05; ‡, p<.01; | | | | | | | |
